# Starch granules in algal cells play an inherent role to shape the popular SSC signal in flow cytometry

**DOI:** 10.1186/s13104-024-06983-6

**Published:** 2024-10-29

**Authors:** Michael Sandmann, Michael Rading

**Affiliations:** 1 University of Applied Sciences Neubrandenburg, Brodaer Straße 2, D-17033 Neubrandenburg, Germany; 2 Department of Theory and Bio-Systems, Max Planck Institute of Colloids and Interfaces, 14424 Potsdam, Germany

**Keywords:** Diurnal rhythm, single-cell analysis, flow cytometry, starch, Lorenz–Mie theory; light scattering

## Abstract

**Objective:**

Flow cytometry (FC) is probably the most important technique for single-cell analysis. It’s precisely, rapid, and suitable for multidimensional single-cell analysis. The commonly used side scatter (SSC) intensity determined by FC is often interpreted as a measure of the internal cellular complexity of cells. In simple terms, the more structured a cell is, the higher the SSC intensity quantified by FC. Nevertheless, most of the studies that support this interpretation are based on data derived from animal or human cell lines and while it is assumed that the results can also be transferred to plant or algal cell lines, the details remain unclear. The objective of the recent work is to clarify the interpretation of the SSC signal from algal cells.

**Results:**

Algal lipid droplets and their starch play an inherent role to shape the popular SSC signal derived from FC. This was shown by a theoretical approach based on Lorenz–Mie theory. These results were supported by experiments with different model cultures of *Chlamydomonas reinhardtii* in which a high linear correlation was observed between the SSC signal and the ‘physical’ starch quantity.

**Supplementary Information:**

The online version contains supplementary material available at 10.1186/s13104-024-06983-6.

## Introduction

Single-cell analysis (SCA) can be used to reveal heterogeneity in cell populations and to understand their single-cell dynamics [1–5]. One of the most important techniques to facilitate SCA is flow cytometry (FC). In flow cytometers, optical properties of cells are rapidly analysed which includes quantification of light scattering, and fluorescence derived from these cells. Light scattering is typically characterised as light intensity at two different scatter angles [6–8]. In addition, fluorescent properties of the cells arise either from externally applied fluorescent probes or intrinsic cellular pigments [6–8].

The first typically analysed scatter angle is called wideangle light scatter, also known as side scatter (SSC). The scatter intensity in this direction can be used as a measure of the internal structuredness of cells. It is known that organelles or other small cellular components contribute to this signal [8–13]. Nevertheless, it is not yet clear how the various cell components contribute in detail to the SSC. The second important scatter angle called forward scatter (FSC) results from light being scattered from the cells in the direction of propagation of the incident light. FSC intensity is often interpreted as a measure for the size of cells. Nevertheless, the FSC/size correlation is partially also affected by the granularity of the cells which may lower this correlation. Most of the knowledge of cellular light scattering properties rely on studies with human or animal cell lines, but it does not necessarily reflect the phenomena in plants or algal cells. Plants and algal cells differ significantly in that they contain numerous starch granules, a rigid cell wall and chloroplasts in which photosynthesis occurs [14, 15]. Fluorescent light, emitted from intrinsic pigments of algal cells can be even used to characterize algal populations in biological oceanography [16–18].

To the best of our knowledge, the recent study is the first of its kind to directly indicate that the starch granule fraction is the major contributor to the SSC signal from algal cells. The evidence relies on an application of Lorenz–Mie theory of light scattering to single particles and simplified model cells including a direct comparison of FC data with reference data from different cell cultures.

### Experimental section

#### Model cells

A wild-type strain of the green alga *Chlamydomonas reinhardtii* (CC1690) was used for this study.

#### Preculture and synchronisation

*Chlamydomonas* cells were precultured and synchronized as previously described [19, 20]. The model cultures studied were light-dark synchronized cultures. Briefly, the cells were continuously aerated with CO_2_ enriched air (2% [v/v] CO_2_) in so-called Kniese tubes, that act as simple bubble column photobioreactors. The cells were synchronized by application of a repetitive light–dark regime (12 h light/12 h dark) and daily dilution with fresh culture media at the end of each dark phase. Daily starting cell concentration was 7 ⋅ 10^5^ cells mL^-1^ [19, 20]. Cultivation was done at 800 µmol photons m^-2^ s^-1^ (high light), 400 µmol photons m^-2^ s^-1^ (medium light), and 200 µmol photons m^-2^ s^-1^ (low light). Light intensity was quantified inside the cell suspension (Quantum Scalar Laboratory Type QSL-2100; Biospherical Instruments Inc., San Diego, USA). The whole procedure was performed a total of six times, including two independent biological replicates per light intensity. Replicates were prepared from different colonies after re-cloning.

#### Quantification of cell number

Cell concentration was measured using a MultiSizer 3 particle counter (Beckman Coulter, Krefeld, Germany). Cells between a diameter of 2.28 μm and 23 μm were counted.

#### Starch quantification

Starch extraction procedure and the subsequent quantification of released glucose monomers were performed as described by Garz et al. (2012) [19]. Briefly, starch granules were isolated and then hydrolysed to glucose by Amyloglucosidase. Glucose was then indirectly quantified by an enzymatic starch determination based on the enzymatic reactions of hexokinase and glucose-6-phosphate dehydrogenase. NADPH which is synthesised stochiometric to the glucose monomers was finally quantified by photometric detection at 340 nm. The resulting starch amount per suspension volume was related to the respective cell concentration which gives finally the starch amount per 1 million cells.

#### Flow cytometry

SCA was done by a commercial CYFLOW SPACE flow cytometer (Partec, Münster, Germany), equipped with a blue 488 nm solid-state laser (20 mW), and with the FLOMAX software version 2.5 (Partec, Münster, Germany). Handling of the device and data analysis was done according to the manufacturer. In total fifty thousand cells were analysed for each sample with a maximum count rate of 1000 counts per second. Gain setting for the SSC photomultiplier tube was 195, with a 3-decade range.

#### Calculated side scatter intensities

The freeware tool MIEPLOT (version 4.6.21) was used to simulate side scatter intensities of single subcellular particles and simplified model cells [21, 22]. Taking the small size of ribosomes into account, the calculation of angular scattering of ribosomes was based on Rayleigh theory of light scattering [21]. For all larger cellular ingredients, calculations were done with Lorenz–Mie theory of light scattering [21]. MIEPLOT is able to plot functions for scattering intensity depending on particle sizes, material properties, and illuminating wavelength. In the recent study spherical particles of various refractive indices (RI) and diameters have been considered (Additional file1: Table S1). Unpolarised light at 488 nm was used for calculations. The contribution of the whole organelle fraction in a model cell was calculated as side scatter intensity multiplied by the number of distinct particles belonging to the organelle fraction.

#### Statistical analysis

For statistical analyses, SIGMAPLOT version 14 (Systat Software GmbH, Düsseldorf, Germany) was used.

## Results

### Theoretical approach

**Fig. 1:**
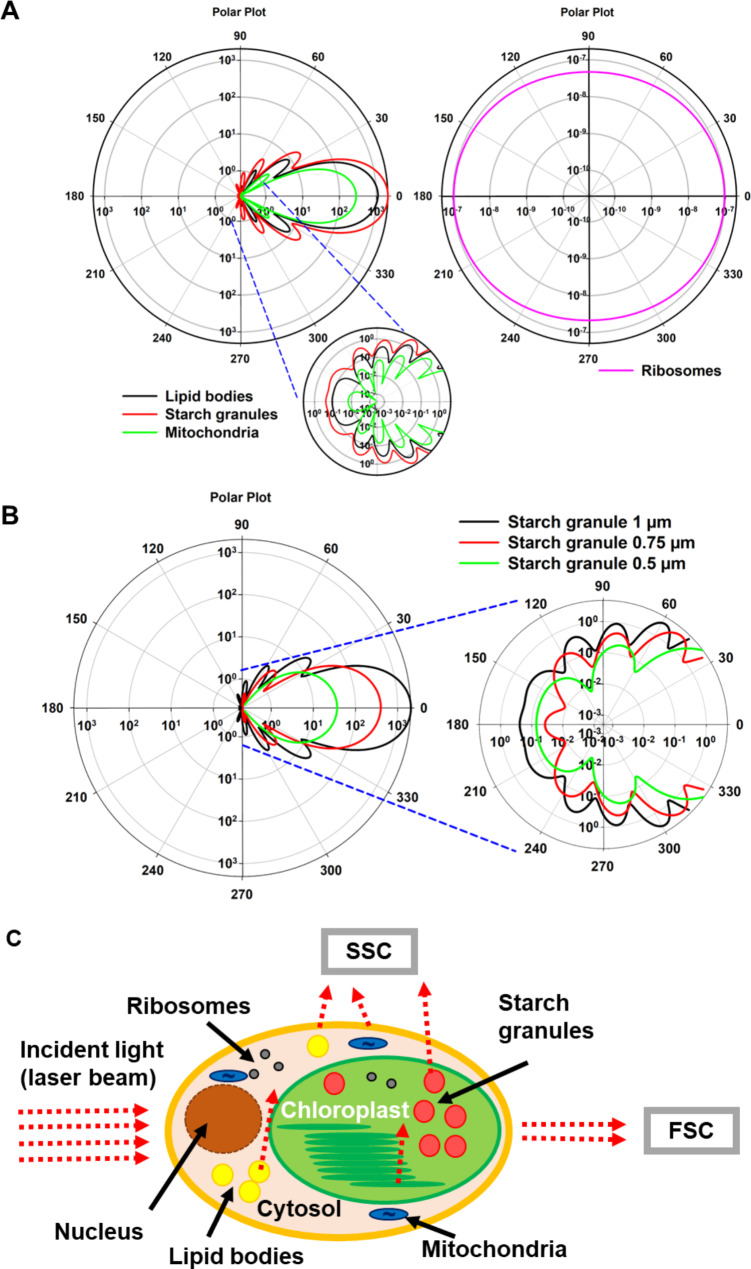
Angle-dependent light scattering. (**A**) Polar plot of scattering angle vs. scattered intensity for single cellular constituents. The insert shows a zoomed-in area. (**B**) Polar plot of scattering angle vs. scattered intensity for starch granules exhibiting different sizes. Insert shows a zoomed-in area. (**C**) Summarized contribution of different cellular constituents in relation to SSC and FSC

Figure 1 shows the angle-dependent light scattering intensity of different cellular constituents. Calculations are done with the MIEPLOT software and based on Lorenz–Mie theory [21]. The resulting visualization can be interpreted as follows, the particles are located in the origin of the diagram (centre of the polar plot), whereas incident light illuminates from the 180° direction, and the calculated SSC intensity can be finally read at 90° or 270°. Mitochondria and ribosomes exhibit much lower SSC intensity than starch granules or lipid bodies. A comparison of different particles at a constant diameter shows that the high RI of lipid bodies and starch cause the high contribution to the SSC intensity (Additional file1: Table S1). For relatively large particles (e.g. 1 μm diameter), most of the light is scattered in forward direction (FSC at 0°) (Fig. 1A). The overall scatter intensity of a ribosome (25 nm diameter) is very low. In contrast to the larger particles, ribosomes show a more isotropic scattering pattern. Finally, the contribution of a single ribosome to the wide-angle light scatter intensity is negligible and the contribution of a single mitochondrion remains comparatively low (Additional file1: Table S1). The relation between the size of starch granules and the calculated scattering intensity is shown in Fig. 1B. During photosynthesis driven starch accumulation in algal cells, the diameter of the starch granules will increase, which leads also to a higher contribution to the SSC intensity (Fig. 1B).

As a simplification, all considerations have been focused so far on scattering events from single particles within the algal cell and the effect of the various cell components remains unclear because, for example, the number of intracellular particles per cell varies greatly between the observed organelle groups. Based on this, Table S2 shows the calculated relative contribution of the expected scattering intensity at 90° (SSC) of the whole organelle fractions in a theoretical model cell. The model cell contained 50 starch granules, 50 mitochondria, 120,500 ribosomes (scenario one) and, if a nitrogen-free media is used, 7 lipid bodies as well (scenario two). The calculation is still simplified because only single scattering events have been assumed (no re-scattering from adjacent particles). As result, most of the scattering intensity at 90° from an algal cell was due to scattering from starch granules (80%), while around 20% originates from the mitochondria fraction and the contribution of ribosomes is negligible (Additional file1: Table S2, Scenario one). Lipid droplets are known to appear almost exclusively in algal cells grown in nitrogen-free media [4, 23]. Even with nitrogen deprivation (Additional file1: Table S2, Scenario two) the contribution of the relatively low amount of lipid droplets per cell will be less than 15% of the total SSC signal.

The theoretical approach showed that the SSC intensity in algal cells is mostly dependent on the starch granule fraction. Figure 1C summarises the contribution of different cellular constituents in relation to SSC and FSC scattering intensity that can be analysed by FC.

### Experimental validation by FC analysis of model cells grown under conditions with different starch content

**Fig. 2:**
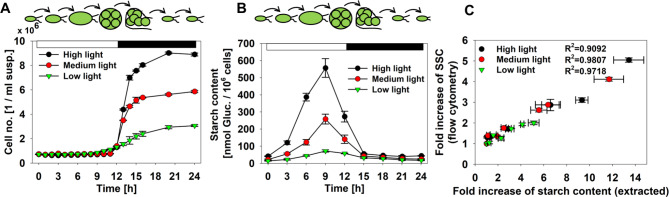
Characterisation of model cultures grown under three different light intensities. (**A**) Cell number based on suspension volume plotted against time. Means ± SD are given (*n* = 4). (**B**) Starch content per 1 million cells obtained by extraction. Developmental cycle of the synchronized algal cells and the duration of the light and dark phase is schematically visualized on top of A and B. (**C**) Correlation of starch content obtained by extraction and mean of SSC signals derived from flow cytometry. Correlation analysis was based on the time points: 0, 3, 6, 9, 12, 15, and 24 h. Development of SSC and starch content was plotted after normalisation. Means ± SD are shown (FC: *n* = 2; extraction: *n* = 4)

The experimental validation of the theoretical results was done using synchronised cultures of the green alga *Chlamydomonas*. Such synchronised cultures are often used as model systems that pass various stages of development more or less in parallel. During their repetitive cellular development, the cells exhibit very different cellular starch concentrations, which can be finally used as a reference for validations. Each synchronised culture is characterised by small cells at a low starting cell concentration at the beginning of each light phase (Fig. 2A). During illumination, the diameter and the cellular starch content of the cells will increase (Fig. 2B). At the end of the light phase cell division and release of the small daughter cells is initiated (Fig. 2A). Release of the daughter cells is usually finalised after a couple of hours (Fig. 2A).

The three different light intensities resulted in different cell number productivities and starch content dynamics and thus can be used as model cultures for a correlation analysis between the SSC signal derived from FC and the starch content determined after extraction. Under the three growth conditions, a high linear relationship between mean SSC signal and ‘physical’ starch content was observed (Fig. 2C). The correlation coefficients are rho = 0.95, rho = 0.99, and rho = 0.99.

## Discussion

FC is a powerful tool for the analysis of cell cultures. Nevertheless, the detailed interpretation of how cells exhibit different scattering intensities in the two typically measured scattering angles from FC devices is often not possible. The reason is that cells are very complex and dynamic structures, which makes accurate predictions of the light scattering behaviour almost impossible. One way of solving this issue is the use of strong approximations. In the past it was shown that there is a correlation between cellular complexity and the SSC-signal [9–13]. Most of the studies are restricted to heterotrophic cells, like human cells. In photosynthetic organisms (including algae) starch granules and lipid bodies seems to be the most important contributors to the SSC intensity that can be easily measured by FC (Fig. 1, Additional file1: Table S1). The reason for this is the very large RI of lipid droplets and especially of starch in comparison to the surrounding medium (approximated as RI from cytoplasm). These storage compounds contribute much larger than ribosomes or mitochondria which should hold true also for other cellular constituents with low RI.

In addition, a more realistic scenario was investigated which includes a realistic number of particles/cellular constituents within a model cell. Also in this scenario, the starch fraction remains the major contributor to the SSC intensity in algal cells (Additional file1: Table S2).

The simplified theoretical approaches were directly supported by experimental comparisons with fast growing model cultures that undergo typical strong developmental dynamics [19, 20, 24]. In a direct comparison between SSC signal from FC and the ‘physical’ starch content within algal cells, a strong linear relationship between SSC signal and starch content was observed (Fig. 2C). The correlation coefficients ranged between 0.95 and 0.99. The cells are grown under three different light intensities resulting in different cellular properties (including e.g. daughter cell productivity, starch content, cell size distributions (data not shown), and chlorophyll content (data not shown)). All the parameters could affect the optical properties of the cells and thus could potentially diminish the expected correlation between SSC Signal and ‘physical’ starch content. Nevertheless, the correlation remains high, and the SSC signal is strongly dependent on the cellular starch content. Consequently, the SSC signal could be interpreted as a strong indicator of the cellular starch content within algal cells and thus could be used for label-free process monitoring in algal biotechnology.

Recent studies have shown that the single cell dynamics of synchronous algal culture are far from being understood [24, 25]. In addition to the present work, alternative FC approaches were established including a combination between FSC and chlorophyll fluorescence analysis or FC-based Raman scattering measurements, allowing relative and label-free starch determinations [26, 27].

It can be concluded that storage compounds with a high RI (starch and lipid droplets) are the most important contributors to the SSC signal typically measured by FC. This enables FC to be used as a sensitive, label-free tool for fast analysis of culture dynamics within algal cell cultures, which finally enables fast and label-free relative starch determinations on a single-cell level.

### Limitations

- As a simplification, only single scattering events were assumed (no re-scattering from adjacent particles).
- In algal cells, the shape of lipid droplets, starch granules, ribosomes and mitochondria are not necessarily spherical and varying sizes have to be considered. Thus, the calculations are only approximations because the Lorenz–Mie theory of light scattering applies to spherical particles.
- Investigation of the interaction of lipid droplets with light was restricted to the theoretical approach. A correlation analysis of cellular lipid amount against SSC was not done experimentally, because the cells are grown under non-limiting conditions. This means the number of lipid droplets is neglectable.

## Electronic Supplementary Material

Below is the link to the electronic supplementary material.

Supplementary Material 1

## Data Availability

The datasets generated during and/or analysed during the current study are available from the corresponding author on reasonable request.

## Abbreviations

FC: Flow cytometry
SSC: Wide-angle light scattering intensity or side scattering signal
SCA: Single-cell analysis
FSC: Scattering in forward direction
RI: Refractive index
