# Supplementary material for: Starch granules in algal cells play an inherent role to shape the popular SSC signal in flow cytometry
Source: BMC Res Notes. 2024 Oct 29;17:327. doi: 10.1186/s13104-024-06983-6 (PMC11523789; doi:10.1186/s13104-024-06983-6)
Supplement: Supplementary file 1 — Supplementary Material 1 [file 13104_2024_6983_MOESM1_ESM.docx]

**Starch granules in algal cells play an inherent role to shape the popular SSC signal in flow cytometry.**

Michael Sandmann^*1^, Michael Rading^2^

^1^University of Applied Sciences Neubrandenburg, Brodaer Straße 2, D-17033 Neubrandenburg, Germany

^2^Max Planck Institute of Colloids and Interfaces, Department of Theory and Bio-Systems, 14424 Potsdam, Germany

**Corresponding author:**

Michael Sandmann

sandmann@hs-nb.de

+49 (0)395 5693-2508

**Table S1: Simulation parameters used and calculated scattering intensity at 90° for single particles (SSC)**

| **Organelle** | **Diameter** | **Reference** | **Refractive index (real part)** | **Reference** | **Refractive index and diameter used** | **Calculated scattering intensity for a single particle at 90° (SSC)** |
| --- | --- | --- | --- | --- | --- | --- |
| Cytoplasm (reference) | **-** | **-** | 1.36–1.375 | Drezek et al. 1999 [5] | **RI = 1.36** | **-** |
| Mitochondria | 0.4–1 µm | Dunn A. & Richards-Kortum, 1996 [1] | 1.38–1.41 | Drezek et al. 1999 [5] | **RI = 1.41**  **d = 1 µm** | **0.061** |
| Ribosome | 25 nm | Manuell et al. 2005 [2] | 1,39 - 1,42 | Wang, 2000 [6] | **RI =1.42**  **d = 25 nm** | **4.68×10^-8^** |
| Lipid body (observed under nitrogen-free conditions) | 0.20–2 µm | Wang et al. 2009 [3] | 1.46 | Jung et al. 2018 [7] | **RI = 1.46**  **d = 1 µm** | **0.233** |
| Starch granule | 0–1.5 µm | Garz et al. 2012 [4] | 1.50–1.54 | Aas,  1996 [8] | **RI = 1.5**  **d = 1.0 µm** | **0.397** |
| Starch granule | 0–1.5 µm | Garz et al. 2012 ]4] | 1.50–1.54 | Aas,  1996 [8] | **RI = 1.5**  **d = 0.75 µm** | **0.234** |
| Starch granule | 0–1.5 µm | Garz et al. 2012 [4] | 1.50–1.54 | Aas,  1996 [8] | **RI = 1.5**  **d = 0.5 µm** | **0.102** |

**Table S2: Calculated scattering intensities of the whole organelle fraction of a single cell at 90° (SSC)**

| **Organelle** | **Estimated number of organelles per cell** | **Reference** | **Scenario one: Calculated relative contribution of the organelles for the scattering intensity at 90° (SSC) [%]** | **Scenario two: Calculated relative contribution of the organelles for the scattering intensity at 90° (SSC) [%]** |
| --- | --- | --- | --- | --- |
| Mitochondria | 50 | Arnold et al. 1980 [9] | **20.72** | **18.03** |
| Ribosome | 120,500 | Lynch et al. 2017 [10] | **0.01** | **0.01** |
| Starch granule | 50 | Garz et al. 2012 [4] | **79.27** | **68.96** |
| Lipid body (observed under nitrogen-free conditions) | 7 | Wang, 2000 [6] | **0** | **13.0** |

**Supplemental references**

1. Dunn A, Richards-Kortum R. Three-Dimensional Computation of Light Scattering From Cells. IEEE J Sel Top Quantum Electron. 1996;2:898–905.
2. Manuell AL, Yamaguchi K, Haynes PA, Milligan RA, Mayfield SP. Composition and structure of the 80S ribosome from the green alga Chlamydomonas reinhardtii: 80S ribosomes are conserved in plants and animals. J Mol Biol. 2005;351(2):266-279. doi:10.1016/j.jmb.2005.06.022
3. Wang ZT, Ullrich N, Joo S, Waffenschmidt S, Goodenough U. Algal lipid bodies: stress induction, purification, and biochemical characterization in wild-type and starchless Chlamydomonas reinhardtii. Eukaryot Cell. 2009;8(12):1856-1868. doi:10.1128/EC.00272-09
4. Garz A, Sandmann M, Rading M, Ramm S, Menzel R, Steup M. Cell-to-cell diversity in a synchronized Chlamydomonas culture as revealed by single cell analyses. Biophys J. 2012;103:1078–86.
5. Drezek R, Dunn A, Richards-Kortum R. Light scattering from cells: finite-difference time-domain simulations and goniometric measurements. Appl Opt. 1999;38(16):3651-3661. doi:10.1364/ao.38.003651
6. Wang RK. Modelling optical properties of soft tissue by fractal distribution of scatterers. J. Mod. Opt. 2000;47:103–120.
7. Jung J, Hong SJ, Kim HB, et al. Label-free non-invasive quantitative measurement of lipid contents in individual microalgal cells using refractive index tomography. Sci Rep. 2018;8(1):6524. doi:10.1038/s41598-018-24393-0
8. Aas E. Refractive index of phytoplankton derived from its metabolite composition, J. Plankton Res. 1996;18:2223–2249.
9. Arnold C, Blank R. 1980. Three-dimensional structure of mitochondria and plastids in Chlamydomonas reinhardii and Polytoma papillatum. In: Schenk H, Schwemmler W, editors. Endosymbiosis and cell biology: A synthesis of recent research. Proceedings of the International Colloquium on Endosymbiosis and Cell Research. Berlin, Boston: De Gruyter; 1980. p. 809-816.
10. Lynch M, Marinov GK. Membranes, energetics, and evolution across the prokaryote-eukaryote divide [published correction appears in Elife. 2018 Jul 05;7:]. Elife. 2017;6:e20437. doi:10.7554/eLife.20437
